# Chemical order-disorder nanodomains in Fe_3_Pt bulk alloy

**DOI:** 10.1093/nsr/nwac053

**Published:** 2022-03-21

**Authors:** Qiang Li, Yang Ren, Qinghua Zhang, Lin Gu, Qingzhen Huang, Hui Wu, Jing Sun, Yili Cao, Kun Lin, Xianran Xing

**Affiliations:** Beijing Advanced Innovation Center for Materials Genome Engineering, Institute of Solid State Chemistry, University of Science and Technology Beijing, Beijing 100083, China; X-Ray Science Division, Argonne National Laboratory, Argonne, IL 60439, USA; Beijing National Laboratory for Condensed Matter Physics, Institute of Physics, Chinese Academy of Sciences, Beijing 100190, China; Beijing National Laboratory for Condensed Matter Physics, Institute of Physics, Chinese Academy of Sciences, Beijing 100190, China; NIST Center for Neutron Research, National Institute of Standards and Technology, Gaithersburg, MD 20899-6102, USA; NIST Center for Neutron Research, National Institute of Standards and Technology, Gaithersburg, MD 20899-6102, USA; Beijing Advanced Innovation Center for Materials Genome Engineering, Institute of Solid State Chemistry, University of Science and Technology Beijing, Beijing 100083, China; Beijing Advanced Innovation Center for Materials Genome Engineering, Institute of Solid State Chemistry, University of Science and Technology Beijing, Beijing 100083, China; Beijing Advanced Innovation Center for Materials Genome Engineering, Institute of Solid State Chemistry, University of Science and Technology Beijing, Beijing 100083, China; Beijing Advanced Innovation Center for Materials Genome Engineering, Institute of Solid State Chemistry, University of Science and Technology Beijing, Beijing 100083, China

**Keywords:** Fe_3_Pt alloy, chemical order, nanodomain, local structure, pair distribution function

## Abstract

Chemical ordering is a common phenomenon and highly correlated with the properties of solid materials. By means of the redistribution of atoms and chemical bonds, it invokes an effective lattice adjustment and tailors corresponding physical properties. To date, however, directly probing the 3D interfacial interactions of chemical ordering remains a big challenge. In this work, we deciphered the interlaced distribution of nanosized domains with chemical order/disorder in Fe_3_Pt bulk alloy. HAADF-STEM images evidence the existence of such nanodomains. The reverse Monte Carlo method with the X-ray pair distribution function data reveal the 3D distribution of local structures and the tensile effect in the disordered domains at the single-atomic level. The chemical bonding around the domain boundary changes the bonding feature in the disordered side and reduces the local magnetic moment of Fe atoms. This results in a suppressed negative thermal expansion and extended temperature range in Fe_3_Pt bulk alloy with nanodomains. Our study demonstrates a local revelation for the chemical order/disorder nanodomains in bulk alloy. The understanding gained from atomic short-range interactions within the domain boundaries provides useful insights with regard to designing new functional compounds.

## Teaser

A direct probe to the 3D interfacial interactions is developed based on the pair distribution function method, which provides the atomic insight to the chemical order-disorder nanodomains in alloy.

## INTRODUCTION

Chemical order refers to the atomic preferred occupation or displacement at spatial sites, providing a dominated near-coordination environment around the specified atoms and new features of chemical bonds [1,2]. Accompanied by the rearrangement of the lattice potential field and spatial channels in crystals [3,4], chemical order has been viewed as a promising approach to tailoring functionalities or exploring new properties [5,6]. For example, with the assistance of the superlattice growth technique, the chemical order of Pr-dopant was introduced into manganite oxides to obtain suppressed electronic phase separation [7]. An improved screening effect between ions was reported during the formation of B2-type order in FeV bcc solid solution, which then reduced the atomic interactions and resulted in abnormal positive vibrational entropy [8].

However, the formation of chemical order strongly depends on the chemical components, synthetic routes and kinetic conditions [9,10]. The different chemical constituents in solid solution determine the bonding difference, which controls the ability of an ordered atomic distribution and lattice symmetry at the equilibrium state [11]. For instance, the ordered occupation of Na atoms on the diagonal sites of the coordination around oxygen will induce the preferred tilt of octahedra in the 2 × 2 × 2 supercell of the relaxor ferroelectric Na_1/2_Bi_1/2_TiO_3_ [12]. The fine tuning of the synthetic condition will lead to different kinetic driving forces and thus transitions of assembling forms from solution, nanodomains to long-range chemical order. A solid solution with completely long-range order usually requires a suitable temperature and long annealing time according to the laws of thermodynamics. Therefore, the short-range-ordered, partial-ordered and nanoscale-ordered domains are the most prevalent forms in chemical ordered solids. For instance, in CuInS_2_ nanocrystals, the interlaced nanodomains with cation order were observed to separate the effect of domain boundaries on the electron and phonon transport, which could be an ideal structure for thermoelectric application [13].

Chemical order can be used as one of the viable approaches to designing band structure, chemical bonding [14,15], lattice strain [16] and magneto-crystalline coupling [17]. However, the atomic spatial insight for the chemical ordering has been a great challenge. The quantitative characterization for the boundary correlation of nanoscale domains is the pivotal basis on which to design chemical order, yet remains mysterious. In bulk alloy systems, the preferred site occupation of atoms in a lattice, derived from the favored bonding between heterogeneous atoms, is the main feature of chemical order, which often results in a stiffer crystal lattice and peculiar orbital hybridization [1,18]. Nevertheless, the intrinsic metallic bonding feature could provide sufficient lattice compatibility and coherent matching during ordering. Understanding the lattice interface of ordered and disordered domains would facilitate the progression of structural deciphering for symmetry and size. Diffraction information, as the rough structural average, is inadequate to reveal the size distribution and spatial correlation of the ordered/disordered constituents. Reconstitution by atomic electron tomography is effective in acquiring a model of a single nanoparticle at atomic level, but inept in the local regions of a bulk alloy due to the projective characteristics [19].

A method for local structural determination, the pair distribution function (PDF), was expected to advance atomic deciphering for the chemical ordered nanodomains. This method has the natural advantage of revealing short-range information. Local commensurate correlation in Bi_2_Mn_4/3_Ni_2/3_O_6_, contributing to the short-range ferroelectric correlations between Bi^3+^ cations within local polar regions of 12 Å [20], was probed by neutron total scattering. Ti chemical order with sub-nanometer scale and the strong distortion of TiO_6_ octahedra were found by reverse Monte Carlo (RMC) from the neutron PDF of the Nd_2_M_2_O_7_-class high-entropy oxides [21]. Even for PtNi alloy nanoparticles with average disordered structure, the hidden local layered ordering as L1_0_ phase was revealed on the surface parts [22].

In this work, we studied the nanosized chemical ordered/disordered domains in Fe_3_Pt bulk alloy and their related property adjustment. From the obvious scattering difference of Fe/Pt atoms seen in the X-ray, the atomic models during chemical ordering were uncovered. The abundant assembling forms of different ordered/disordered phases of Fe_3_Pt and their similar lattice parameters facilitate exploration of the lattice matching between order/disorder nanodomains. 3D details of chemical ordered/disordered nanodomains at the single-atom level were decoded quantitatively through combined PDF and RMC. From the insightful information on the domain interfaces and lattice strain, the adjustment of the nearest coordination, lattice thermal expansion and magnetic-related property were elaborately analyzed. Our study provides guidelines for designing chemical ordering and quantitative 3D details of nanodomains on the atomic level in bulk alloys.

## RESULTS AND DISCUSSION

### Adjustment of chemical order in Fe_3_Pt bulk alloy and average structure

**Figure 1.**
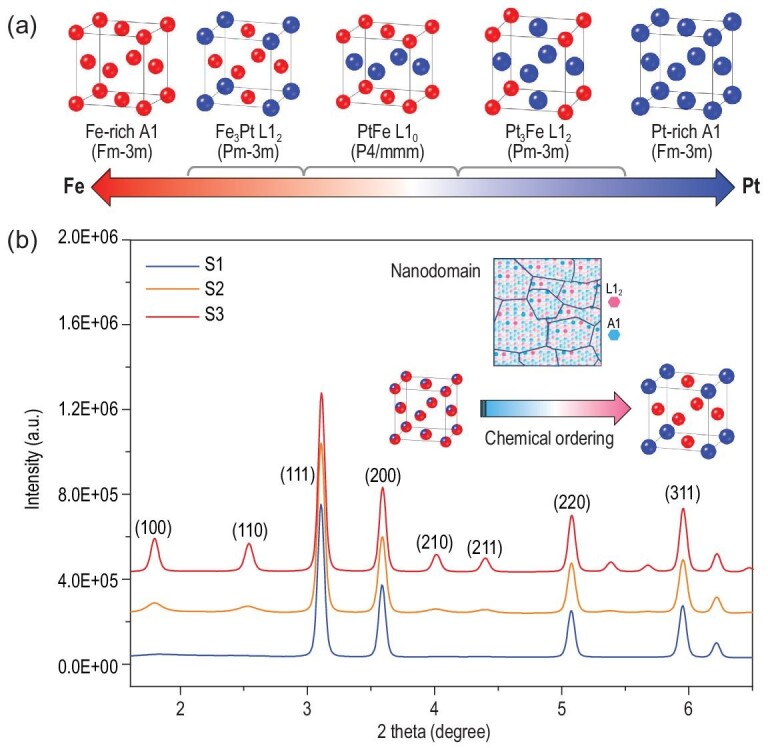
(a) The transition of crystal structure with the chemical component in Fe-Pt alloy. (b) X-ray diffraction of Fe_3_Pt alloy under different annealing conditions (S1: 550°C, 2 h; S2: 650°C, 10 h; and S3: 650°C, 48 h). Inset is the graphical representation of nanodomains in bulk alloy.

With a change of chemical components, Fe-Pt alloy can form three types of similar chemical ordered structures [19]. Starting from the original face-centered cubic (FCC) structure, A1 (Fig. 1a), the preferential occupation of Fe or Pt atoms at the corners of the cubic cell lowers the crystal symmetry from Fm-3m to Pm-3m named L1_2_ phase. Layered stacking of Fe and Pt along the [001] direction will induce a shrinkage along the *c* axis, and further lower the symmetry to P4/mmm, resulting in the L1_0_ phase. The Fe-Pt phase diagram indicates the medium temperature range for the existence of chemical ordered phases. Suitable selection of an annealing condition could best control the degree of chemical ordering in Fe_3_Pt bulk alloy [23]. After the four-time arc remelting of the samples containing 99.99% high-purity metals had been conducted four times, an initial disordered ingot was obtained by water quenching. Three different annealing conditions (S1: 550°C, 2 h; S2: 650°C, 10 h; and S3: 650°C, 48 h) across the ordering temperature range were adopted in order to tune different chemical ordering and assembling forms. Inductively coupled plasma (ICP) test confirmed the accurate chemical composition as being Fe_77_Pt_23_, near the Fe_3_Pt L1_2_ phase region, indicating a possible ordered structure with preferred Pt occupation at the corners of the cubic cell.

X-ray diffraction (XRD) on the Fe_3_Pt samples demonstrates the transition of the average structure from chemical disorder to order at different annealing conditions (Fig. 1b). S1 displays the diffraction peaks of (111), (200), (220) and (311), indicating that the Fe_3_Pt sample shows a similar structure as γ-Fe with the completely random occupancy of Fe/Pt atoms. With increasing temperature, the corner sites in the Fe_3_Pt cubic cell are no longer equivalent to the face-center sites. The characteristic peaks of chemical ordering such as (100), (110), (210) and (211) can be found in S2 and S3, indicating the increased degree of chemical order. Taking the ratio of the peak area of (100) to (200) to evaluate the chemical ordering degree [24], we can determine the average chemical ordering as 0%, 24% and 57% from S1 to S3. The change of peak width of (100) and (110) during chemical ordering can be used to identify the growth of the nanosized aggregation of ordered cells.

### Order-disorder nanodomains in Fe_3_Pt bulk alloy and atomic-level insights

**Figure 2.**
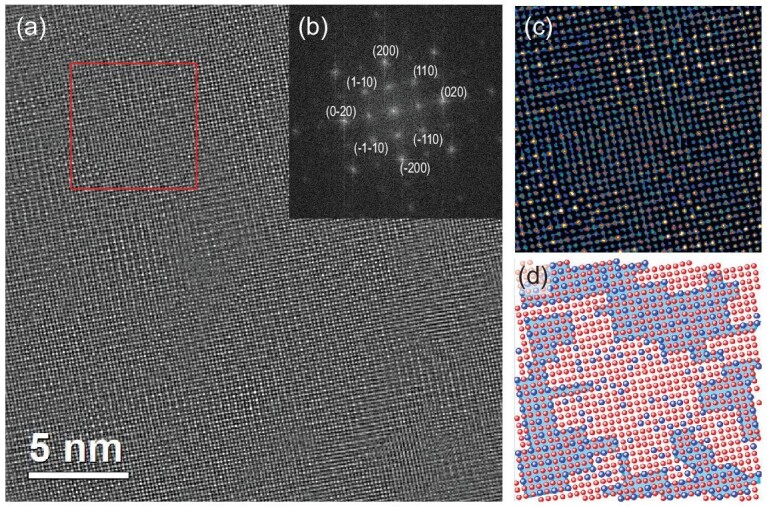
(a) HAADF-STEM image of S3 along the [001] direction. (b) Fourier transform of (a). (c) Enlarged colored view for red-framed area in (a). (d) The sketch map of atomic distribution. Fe and Pt atoms are colored as red and blue balls respectively.

In order to clarify the real distribution of the disordered/ordered components during chemical ordering, a high-angle annular dark field-scanning transmission electron microscopy (HAADF-STEM) image of S3 along the [001] direction was conducted (Fig. 2a, Fig. S1). In a single crystal grain, nanosized domains of chemical ordered-disordered arrangement were clearly observed embedding with each other. The thickness of the sampling area in this image is roughly dozens of nanometers according to the preparation method of ion milling. The rough recognition of the ordered/disordered unit cell in the projected image is convincing. The aggregation scale of the number of ordered cells ranges from a few to dozens. Since the chemical ordering process is controlled by kinetics, the average chemical composition remains homogeneous. Similar constituents in the ordered/disordered domains are beneficial with regard to restricting lattice mismatching on the boundary, and thus could stabilize the coherent interface mode like nanodomains. In the Fourier transformation of the HAADF-STEM image (Fig. 2b), {200} spots were demonstrated with the focused feature while {100} were displayed as diffuse spots. Despite the macro average view of a single crystal, the different convergence between the ordered (100) spots and the disordered (200) confirms the interlacing of the coherent precipitates consisting of ordered-disordered nanodomains. According to the Z^2^ relation of image intensity with atomic number [25], the ordered structure can be preliminarily recognized according to the atomic rows at the corner sites (Fig. 2c). For the completely disordered A1 structure, the atom columns on all sites contain 1/4Pt and 3/4Fe. While in the ordered L1_2_ structure, the corner sites only contain Pt while the face-center sites are dominated by Fe atoms. This is the basis for the rough recognition of atomic distribution in the HAADF image. In Fig. 2d, nanoscale ordered/disordered domains within the range of several or dozens of unit cells exhibit island-shape insertion. Calculated from the distribution of Fe/Pt atoms in this image, the composition is verified as 21.8% Pt, which is close to the ICP result. Under the same lattice orientation, perfect lattice matching can be observed.

**Figure 3.**
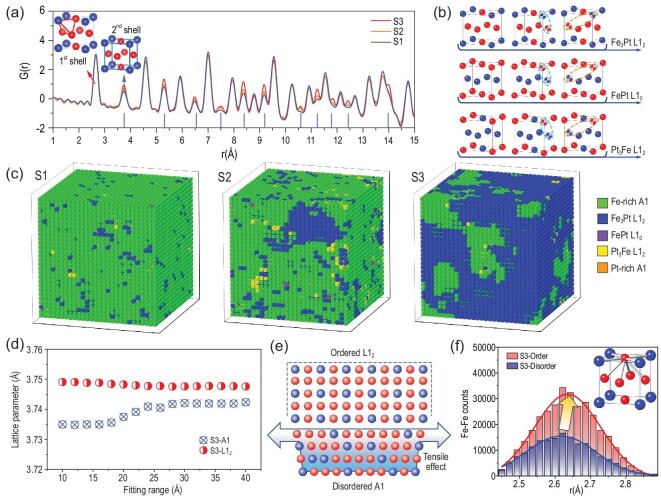
(a) PDF of the Fe_3_Pt bulk alloy. The inset represents the first (red rod) and second (blue rod) shells in the chemical ordered structure. The blue marks at the horizontal axis indicate the positions of the enhanced Pt sublattice in the ordered structure. (b) Standard structure and anti-site defects in the ordered structure of Fe-Pt alloy during the analysis of local cells. (c) Distribution of lattice units according to RMC for PDF of Fe_3_Pt alloy at -100°C and the stripping of nanodomains. Colored cubes stand for the different unit cells with green, blue, purple, yellow and orange colors for disordered Fe-rich A1, Fe_3_Pt L1_2_, PtFe L1_0_, Pt_3_Fe L1_2_ and Pt-rich A1 respectively. (d) Lattice variation according to the fitting range in PDF combining A1 and L1_2_ phases in S3. (e) Interfacial tensile strain around the ordered/disordered nanodomains boundary. (f) Comparison of Fe-Fe pair counts between the ordered and disordered domains in S3 around first coordination shells. Insets are the corresponding bonds in one unit cell.

Even so, further revelation of the relation between the local unit cells and the interface interaction is difficult based on these results, which look forward to a local structural determination method for pair distances and 3D distribution. Due to the magnetic and structural transformation at the high temperature range, PDF was conducted at -100°C. According to the atomic pair peaks in PDF, alternatingly enhanced pairs from the distinct Pt sublattice can be verified as being due to the increase of chemical ordering (Fig. 3a). During the quantification of the contributions from different nanodomains, RMC for the PDF and the subsequent local-cell stripping were adopted [26,27]. Considering the size of the chemically ordered domains and statistical accuracy, RMC simulations were conducted on the 30 × 30 × 30 supercell models with a size of ∼112 × 112 × 112 Å, containing 83 160 Fe and 24 840 Pt atoms.

Reasonable local structural models with 3D atomic arrangements can be attained through the best fitting of the observed PDF patterns (Figs S2–4). Before the analysis by the RMC model, the other nine simulations were conducted starting from the different origin disordered structures. As shown in the partial PDF and structure models (Fig. S5), all the structures have the same local-range structural feature and similar distribution of the disordered/ordered nanodomains. It confirms the reliability and universality of our RMC model in describing local structure. In order to distinguish the local symmetries of different unit cells, the atomic occupation at the corner or the face-center sites should be considered respectively [19]. Due to the atomic solubility over the stoichiometric proportion of the ordered structure, one anti-site defect at the corner or face-center sites can be tolerated during the evaluation of the ordered cells (Fig. 3b). Results from RMC well reproduced the distribution of the ordered/disordered nanodomains and the domain sizes from high resolution transmission electron microscopy (HRTEM), supporting the further quantitative evaluation of the local lattice (Fig. 3c). For the S1 sample, the disordered Fe-rich A1 cell accounts for the box model more than the sporadic distribution of ordered cells. With an increasing degree of chemical ordering, ordered cells gradually aggregate together to form the nanosized domain containing roughly tens of cells. As for S3, ordered domains gather with each other, segmenting the disordered matrix into islands.

The lattice interaction around the domain boundary is the media transferring the lattice vibration, bonding, magnetic behavior and orbital hybridization during chemical ordering. When the average lattice fitting combining A1 and L1_2_ for PDF was applied, the lattice variation in S3 could be confirmed (Figs S6–8). With the increase of fitting range, atomic pairs located on the surface of nanodomains, which possess larger pair distances, contribute more weight to the PDF. As shown in Fig. 3d, the lattice of the unit cell in the disordered domains demonstrated a clear tensile effect while those in the ordered domains remained almost unchanged. This means the ordered L1_2_ cells act as active components to aggregate around the order/disorder interface while the disordered domains are the passive ones (Fig. 3e). This is the result of the stronger chemical bonding between heteroatoms in the ordered phase and thus the stiffer lattice compared to the disordered phase [1]. Chemical order not only introduces a new-coordination environment to the ordered domains but also provides a widespread chemical pressure around the ordered/disordered interfaces. Stripped from the RMC, Fe-Fe pairs in the ordered domains of S3 provide a pair strain of 0.7% on those in the disordered domains, which will significantly affect related functionalities.

### Property tailoring derived from order-disorder nanodomains in Fe_3_Pt bulk alloy

**Figure 4.**
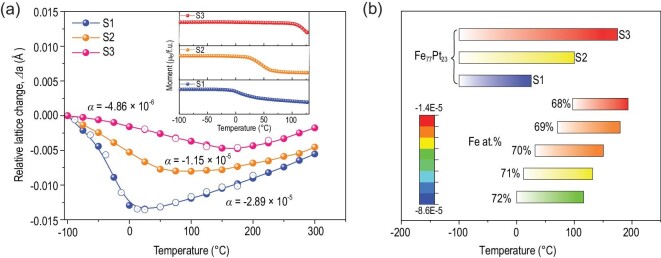
(a) Relative lattice change of Fe_3_Pt alloy from Lebail fittings of XRD patterns. Filled and unfilled circles indicate the first and second cycle tests respectively. The inset shows the temperature dependences of magnetization (M) at a magnetization field of 100 Oe for bulk Fe_3_Pt alloys. (b) A comparison of the NTE performance of the as-prepared Fe_3_Pt alloy in the present study with the results previously reported using chemical composition tailoring [28]. The colored bar is the coefficient of volumetric thermal expansion.

Invar behavior is one of the most important properties of Fe_3_Pt alloy because of its unusual existence in both ordered and disordered states [28]. The disordered state is ferromagnetic at room temperature [29]. However, the ordered L1_2_ Fe_3_Pt has a high Curie temperature *T_c_* of 137°C [30]. In the layered-stacking L1_0_ FePt, the *T_c_* improves to 477°C [31]. L1_2_ Pt_3_Fe is paramagnetic at room temperature but was reported with two types of antiferromagnetic structure below -110°C [32,33]. On account of the hybridization among the Fe 3d, Pt 5d and free-electron-like sp states in the itinerant-model of Fe-Pt alloy, the magnetic moments of Fe are controlled by the lattice parameter [34,35] and chemical ordering. A large lattice constant usually means a high moment [36]. At the same time, the increase of chemical ordering provides the possibility to obtain the enhanced exchanging splitting and atomic magnetic moments [37]. The Lebail fitting of XRD patterns demonstrated the extension of the lattice negative thermal expansion (NTE) under the adjustment of the ordered/disordered nanodomains (Fig. 4a, Fig. S9). In accordance with past results, the chemical ordering brings the larger lattice parameter of the average unit cell at 300°C. The lattice parameter of S3 at such temperature is 3.73630(1) Å and for S1 it is 3.73366(1) Å. It confirms the pure contribution of chemical ordering without magnetic ordering above *T_c_*. When it comes to the ferromagnetic state, i.e. at -100°C, the complex magneto-crystalline coupling between the ordered/disordered interface makes the lattice parameter get close to each other. The emerging ordered nanodomains improved the transition temperature from NTE to positive thermal expansion (PTE) [38], but diminished the sharp NTE coefficient. This kind of NTE in magnetic alloys is closely related to the magnetic phase transformation. According to the M-T curves (inset of Fig. 4a), the *T_c_* of different samples are in excellent accord with the transition points of their corresponding NTEs determined from the relative lattice changes. However, the diffuse NTE behaviors were not accompanied by the diffuse magnetic transition. The existence of the chemical ordered/disordered nanodomains in S3 significantly suppressed the sharp lattice contraction as in S1 and expanded the temperature range of NTE. As previously reported [28], chemical composition could change the position of *T_c_* effectively but confine the NTE in the range of 100°C from the magnetic transformation temperature (Fig. 4b). The presence of the order/disorder nanodomains in Fe_3_Pt alloy could simultaneously suppress the sharp lattice diminution with the increase of temperature as well as extend its temperature window to 250°C from the magnetic phase transition temperature.

**Figure 5.**
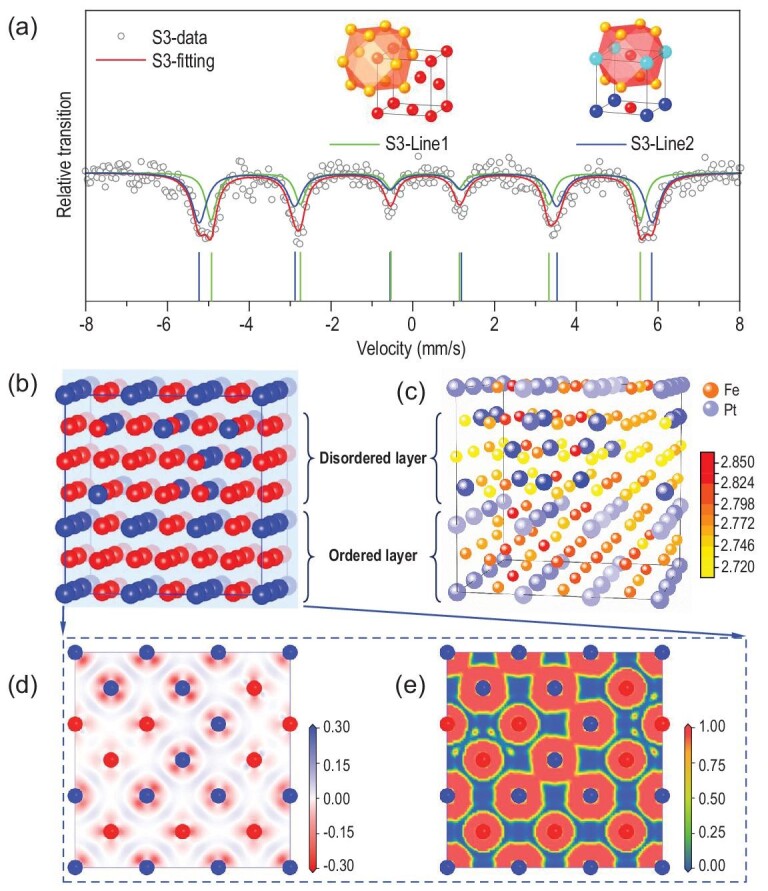
(a) ^57^Fe Mössbauer spectra of as-prepared Fe_3_Pt alloy S3. The blue line shows the higher hyperfine field while the green line shows the lower one. Marks show the positions of the fitted peaks. (b) Domain boundary model of the first principles calculation of Fe_3_Pt alloy. Fe atoms are red while Pt are blue. (c) The calculated atomic magnetic moments. The unit is μ_B_. The bigger blue balls represent Pt atoms and the smaller yellow and orange ones are Fe atoms mapped by the local magnetic moment. (d) Electron density difference and (e) electron localization function around the domain boundary in Fe_3_Pt alloy.

From the fittings of the neutron diffraction patterns on the magnetic structure (Figs S10–11), both ordered and disordered samples show the ferromagnetic state within the NTE range. From the view of the near shell, the local magnetic moment of Fe plays a decisive role in the invar behavior [39]. Due to the interlacing of A1 or L1_2_ nanodomains, the nearest neighbors in the coordination environment around Fe atoms can mainly be classified into two types. One is the random distribution of Fe/Pt in the 12 coordination sites, containing 9Fe and 3Pt in total, according to the chemical composition in A1 phase (inset of Fig. 5a). The other is the ordered distribution of Pt/Fe with the preferred occupation of 4 Pt at the corner sites of a cubic cell and 8 Fe at the face centers. The large difference between the magnetic moment of Fe and Pt leads to a change of the local magnetic field [40–42]. The ^57^Fe Mössbauer spectrum of S3 (Fig. 5a, Table S1) reveals that there are two lines corresponding to the local environments in the ordered and disordered nanodomains. Based on the degree of chemical order, ∼57% determined from XRD and local cell extraction in RMC, the more weighted line 2 stands for the situation in L1_2_ nanodomains, while line1 stands for A1. The ratio of the local environments shown in Table S1 is in agreement with the degree of order determined from XRD. Generally, the substitution of Pt by Fe in the nearest neighbor coordination of the disordered unit cell will enhance the local magnetic field on account of the large moment of the Fe atom. However, fitting results in the Mössbauer spectrum confirm the lowered ultrafine field of line1 as shown by the marked distance. The decrease of Fe moments in disordered domains leads to a reduced magnetic contribution to magnetostriction on the spontaneous volume and thus a moderate NTE behavior.

In order to gain insight into the structural origin, the first principles calculation on the domain boundary model was conducted. In a 3 × 3 supercell (shown in Fig. 5b) with the size adopted from the lattice parameters of an ordered cell, the lower and upper halves of the supercell are constructed as the ordered and disordered sides around the boundary of an ordered/disordered domain. For the completely disordered model of the same size, the average magnetic moment of Fe atoms was calculated as 2.74μ_B_, greater than that in the ordered model, i.e. 2.68μ_B_ (Table S2, Fig. S12). This is consistent with a previous report [39]. However, around the domain boundary, a suppressed Fe moment was obtained in the disordered side compared to the ordered side (Fig. 5c), reproducing the behavior of a reduced local magnetic field in the ^57^Fe Mössbauer spectrum of S3. In the electron density difference result (Fig. 5d), both Fe and Pt provide the itinerant electrons between the atoms. But the increase in the number of Fe-Fe pairs in the disordered side reduces the number of itinerant electrons. Corresponding to the electron localization function (Fig. 5e), in which the value of 1 represents the localized electrons and 0 indicates the free electrons, the change of local coordination environment and tensile effect on the disordered lattice leads to the generation of localized electrons with restricted extent in the middle of homogeneous atoms. This would cause a decrease of the local magnetic moments of Fe atoms in the disordered domains and thus the suppression of NTE in Fe_3_Pt bulk alloy with chemically ordered/disordered nanodomains.

## CONCLUSIONS

In summary, our work provides insights on the 3D atomic level for Fe_3_Pt bulk alloy with chemically ordered/disordered nanodomains. RMC simulation from PDF data elaborates the structure observed in the HAADF-STEM image and the quantitative spatial distribution of the local unit cells. The nanosized domains with chemical order/disorder on a scale of tens of unit cells play a decisive role in property adjustment. Detailed evaluation of the interfacial lattice around the boundary reveals the tensile effect on the disordered side. As the passive component, the lattice strain and the change of coordination environment of Fe atoms in the disordered domains restrain the itinerant feature of the conduction electron, leading to reduced local magnetic moments of Fe atoms in the disordered domains. The present experimental and theoretical results for the identification of local structures in nanodomains, and the lattice matching around interfaces, provide a convincing structural understanding and establish an effective chemical approach to tailoring the NTE properties of bulk alloys.

## METHOD

### Synthesis

Fe_3_Pt alloy was prepared by arc melting using raw metal with 99.99% purity. After remelting four times, the ingot was annealed at 900°C in a sealed quartz tube in a vacuum condition for 4 days. Then the ingot was quenched in water and ground to a fine powder. In order to get samples with different chemical ordering, the powder was annealed again under the same condition as before (S1: 550°C, 4 h; S2: 650°C, 10 h; S3: 650°C, 48 h).

### Characterization

The Fe_3_Pt alloy powder, after sieving, was dispersed in ethanol and then dropped on a carbon-coated copper grid for HAADF-STEM (JEM-ARM 200F). The crystal structures were confirmed by XRD. High-T experiments were conducted on PW 3040-X’Pert Pro, PANalytical, Cu Kα. Room temperature (RT) experiments used for Lebail fitting were conducted by synchrotron XRD of beamline 11-ID-C in advanced photon source (APS) with wavelength 0.1173 Å. The Lebail fitting was conducted by FullProf [43]. Atomic PDF was obtained from high-energy X-ray scattering data by direct Fourier transform of reduced structure function with Q value of 26 Å^–1^ by program PDFGETX2 [44], using the BL08W beamline at SPring-8 with a wavelength of 0.1078 Å. Average fittings were conducted based on PDFgui [45]. RMC for the PDF data was carried out by RMCprofile [46] based on the 30 × 30 × 30 supercell model with initial disordered elemental occupation. DISCUS [47] was utilized to analyze the local coordination environment and distribution. In order to facilitate the extraction of symmetry, the surficial unit cell of the big-box model was excluded during the analysis of the local cell. Magnetism properties were measured on the Superconducting Quantum Interference Device (SQUID) under a magnetic field of 100 Oe. Neutron diffraction experiments were carried out on the nanoscale-ordered materials diffractometer (NOMAD) of Oak Ridge National Laboratory (ORNL). The magnetic structure was extracted from the Rietveld refinement of GSAS-II [48].

### Calculation

First principles calculations based on the projector augmented wave (PAW) were performed using the Vienna *ab initio* simulation package (VASP) [49]. Structures used were extended to a 3 × 3 × 3 supercell. The ordered structure was obtained from the PDF fitting. The disordered model was constructed by random substitution of Fe by 25% Pt in the Fe FCC structure with the same lattice size as the ordered L1_2_ phase. Under the Perdew–Burke–Ernzerhof exchange-correlation functional [50] within the generalized gradient approximation [51] (GGA), the cut-off energy for the basis set was chosen as 270 eV. Initial magnetic moments for Fe atoms and Pt atoms were set as 4 μ_B_ and 0.0 μ_B_ respectively.

## Supplementary Material

nwac053_Supplemental_File
