# Supplementary material for: Chemical order-disorder nanodomains in Fe3Pt bulk alloy
Source: Natl Sci Rev. 2022 Mar 21;9(12):nwac053. doi: 10.1093/nsr/nwac053 (PMC9905646; doi:10.1093/nsr/nwac053)
Supplement: nwac053_Supplemental_File [file nwac053_supplemental_file.docx]

**Supporting Information**

Chemical order-disorder nanodomains in Fe_3_Pt bulk alloy

*Qiang Li^a^, Yang Ren^b^, Qinghua Zhang^c^, Lin Gu^c^, Qingzhen Huang^d^, Hui Wu^d^, Jing Sun^a^, Yili Cao^a^, Kun Lin^a^ and Xianran Xing^*,a^*

a. Beijing Advanced Innovation Center for Materials Genome Engineering, Institute of Solid State Chemistry, University of Science and Technology Beijing, Beijing 100083, China.

b. X-Ray Science Division, Argonne National Laboratory, Argonne, Illinois 60439, United States.

c. Beijing National Laboratory for Condensed Matter Physics, Institute of Physics, Chinese Academy of Sciences, Beijing 100190, China

d. NIST Center for Neutron Research, National Institute of Standards and Technology, Gaithersburg, Maryland 20899-6102, USA

**Results and discussion**


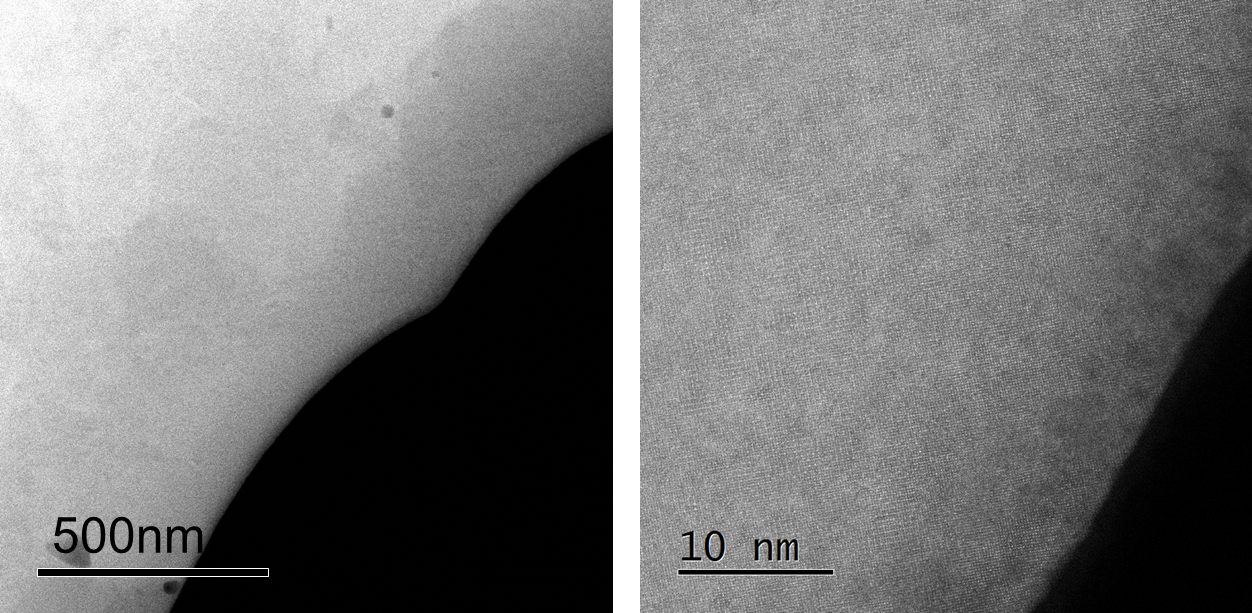


Figure S1. HRSTEM image for S3 with different magnification. After the ion thinning, the edge of the grain shows some obvious thin area. In the amplified view on the right side, the lattice fringe demonstrates the highly integrity in the whole grain. The HAADF-STEM image in Figure 2 is collected in the edge area.


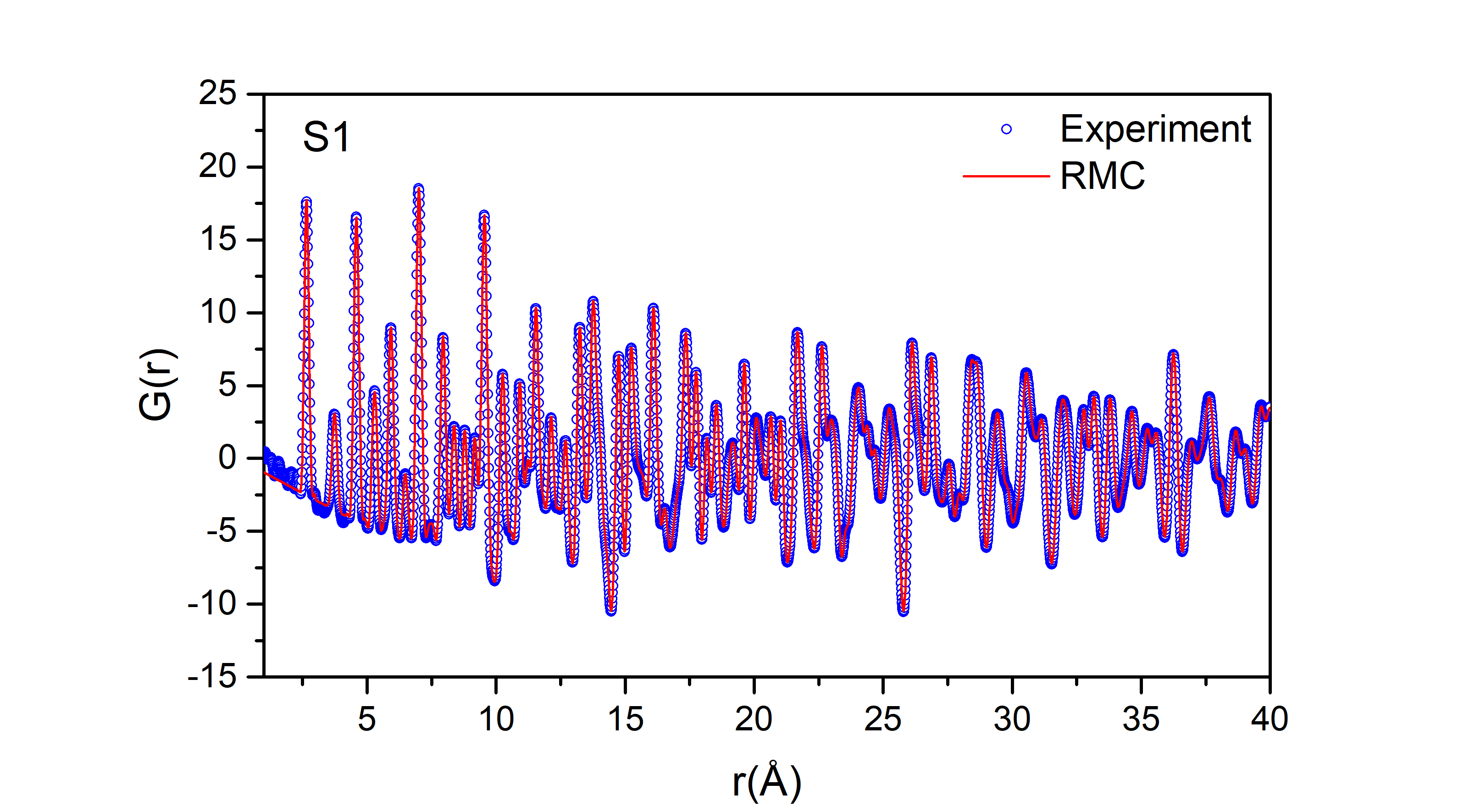


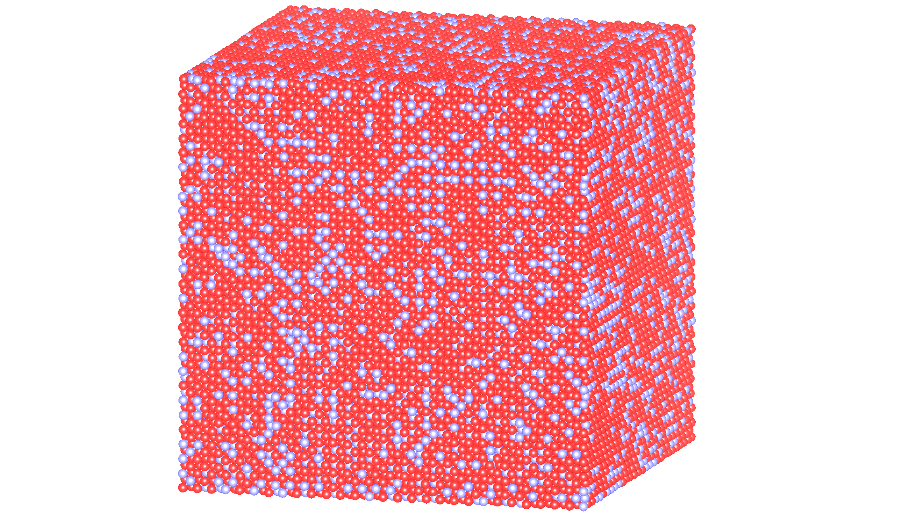


Figure S2. Reverse monte carlo simulation for the PDF of S1 and the atomic model. The starting model is the 30×30×30 supercell with disordered atomic distribution. The simulation time was chosen as 48h. At the end of RMC, the value of chi^2^ reaches 11.5.


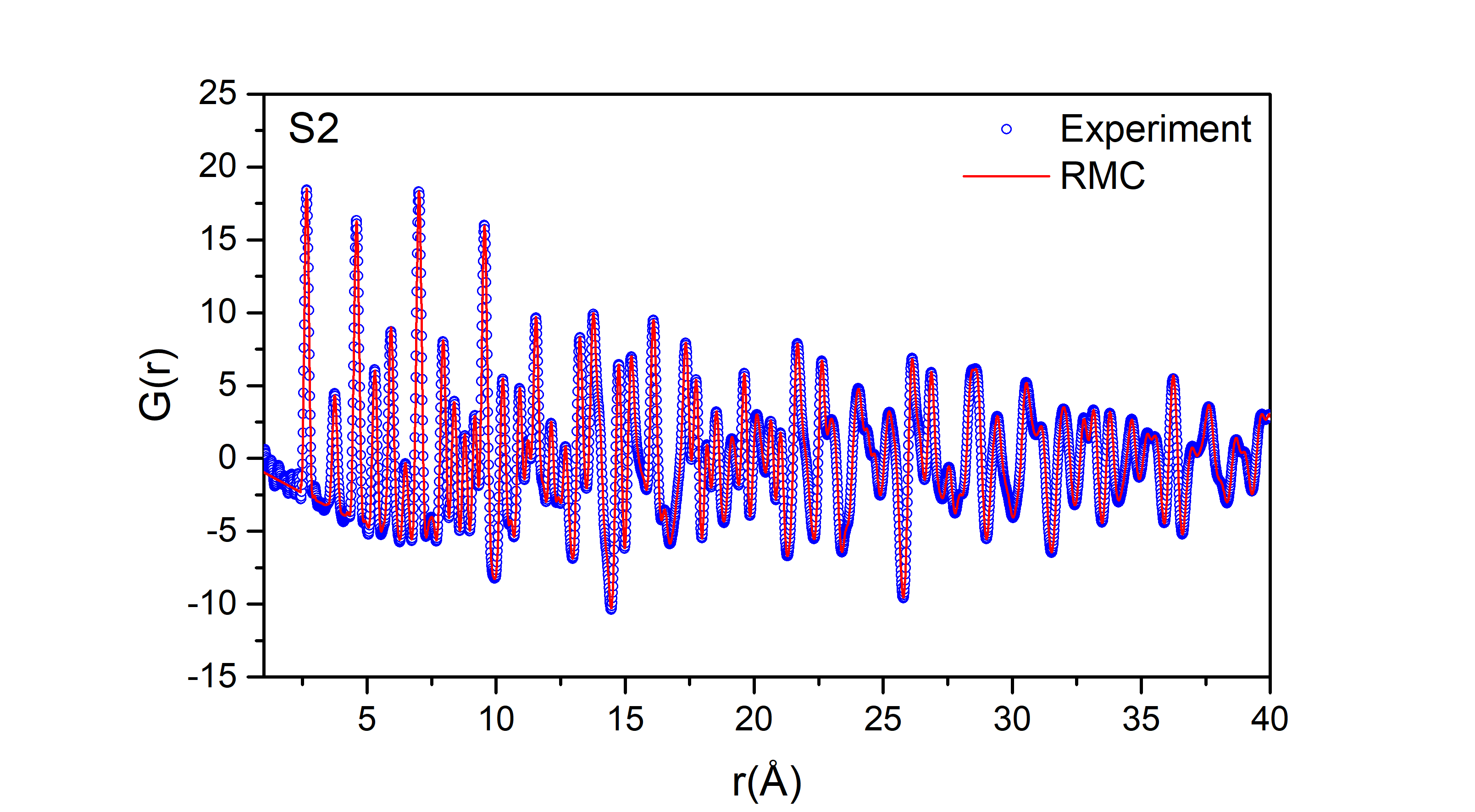


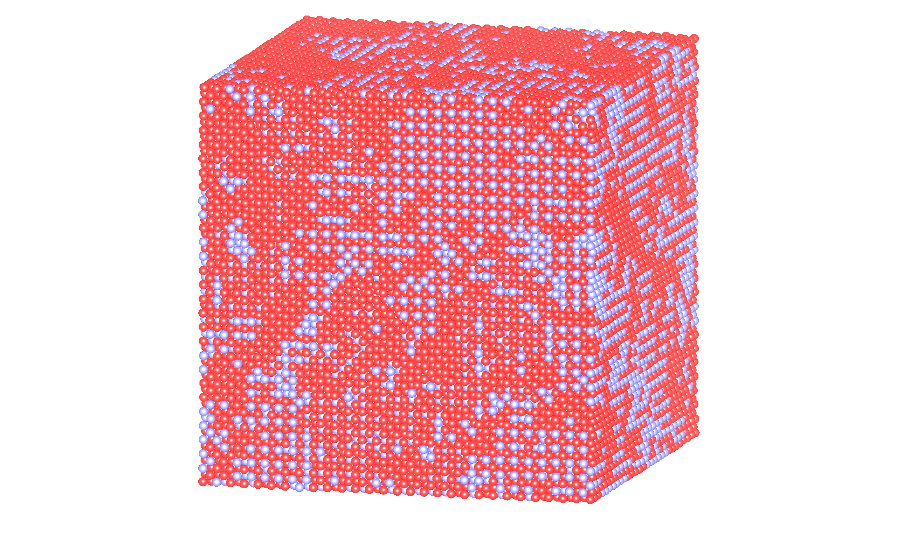


Figure S3. Reverse monte carlo simulation for the PDF of S2 and Atomic model. The starting model is the same with S1. After the RMC simulation of 48h, the value of chi^2^ reaches 9.8.


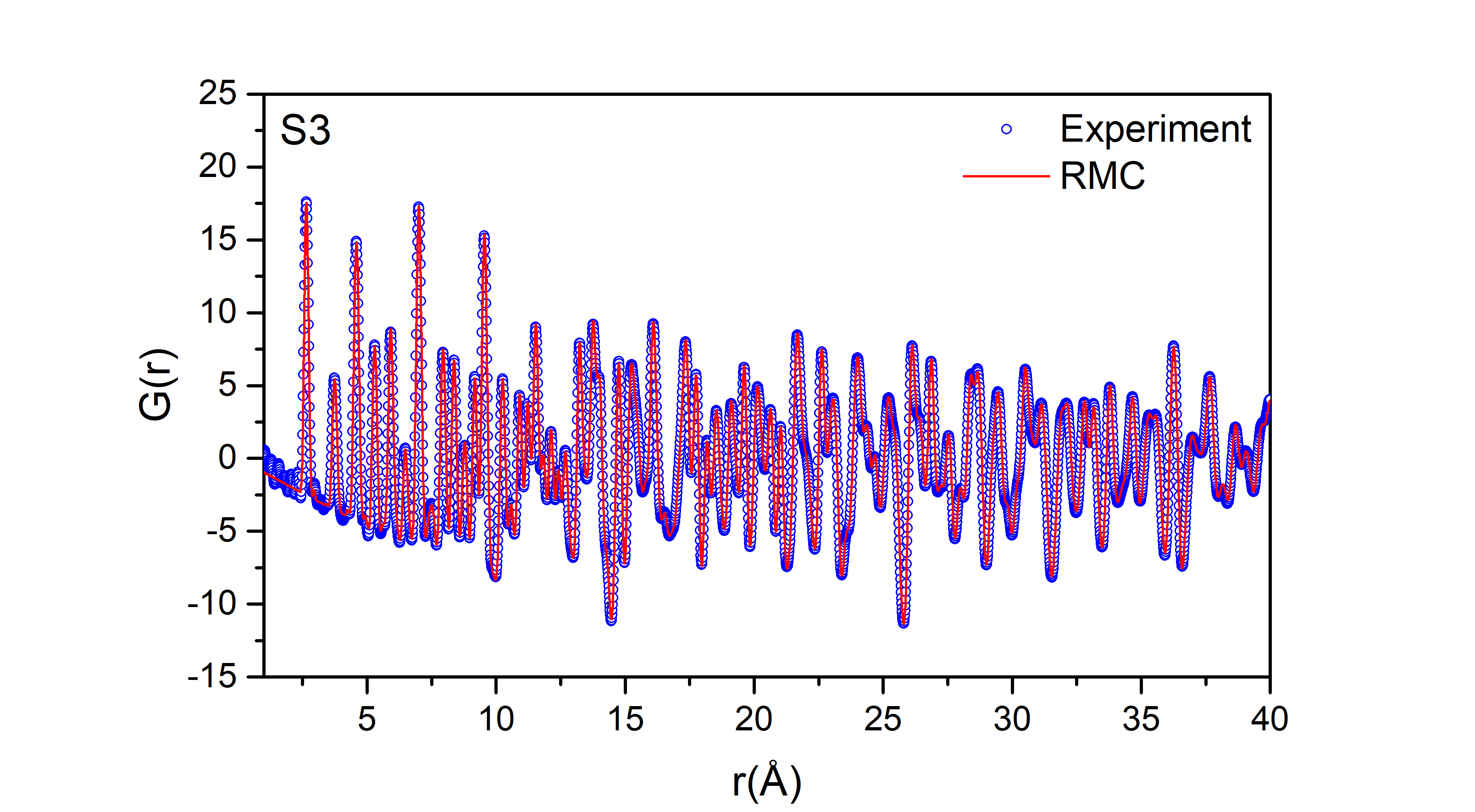


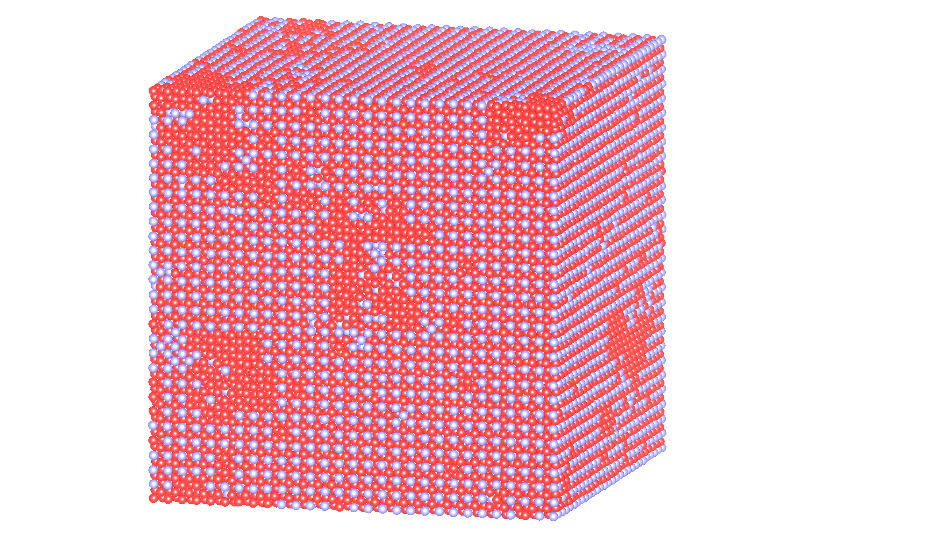


Figure S4. Reverse monte carlo simulation for the PDF of S3 and Atomic model. The value of chi2 after 48h RMC is 13.2.


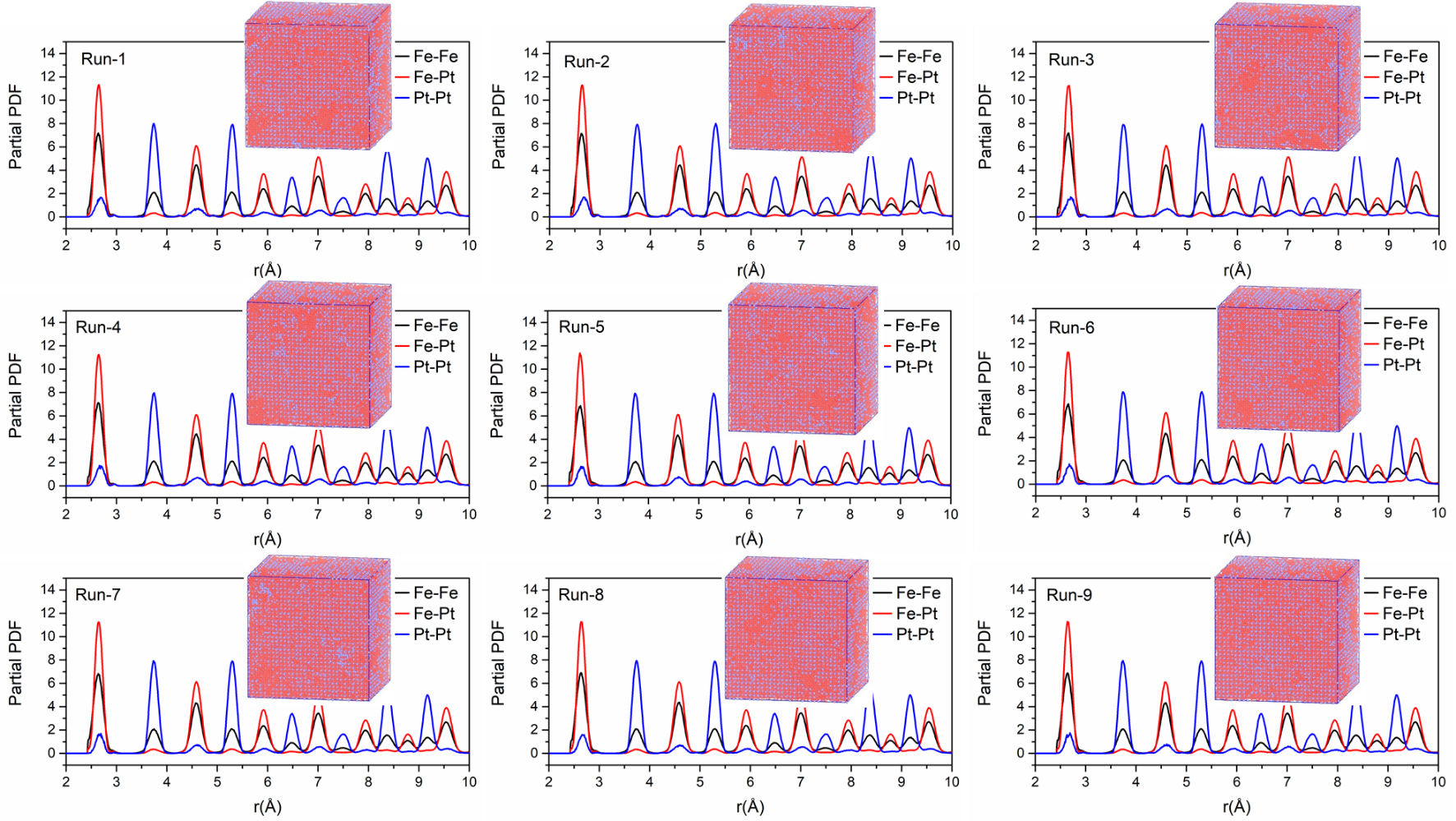


Figure S5. The partial PDF and models from the other RMC simulations of S3 based on the different origin model. All the RMC simulations were conducted with the setting of running time of 48h. The similar domain size and pair distribution in the partial PDF confirm the reliability and consistency of the extraction of local structural features.





Figure S6. Small-box fitting on the PDF of S1 based on A1 unit cell. The perfect consistency between the observed and calculated data verifies the disordered structure of S1 in the local range.


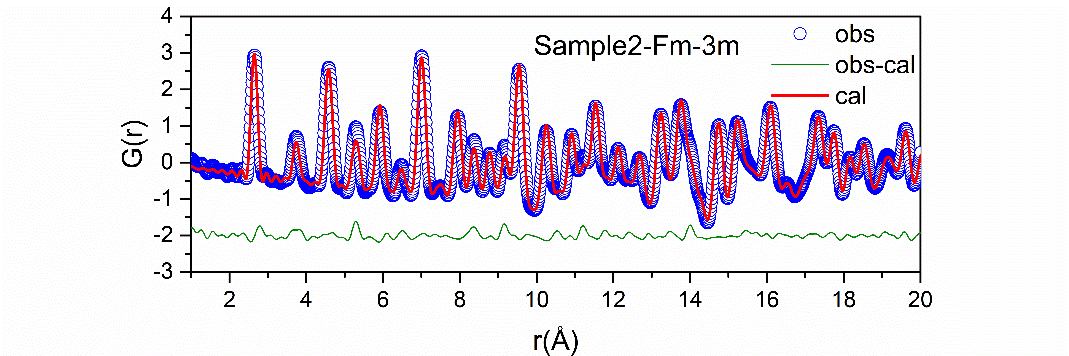


Figure S7. Small-box fitting on the PDF of S2 based on A1 unit cell. Blue boxes show the ordered peaks. Different from S1, some atomic peaks marked by the blue box can’t be described by the disordered Fm-3m structure. They are the sign of ordered unit cells in S2.





Figure S8. Small-box fitting on the PDF of S3 based on L1_2_ unit cell. The fitting based on the ordered Pm-3m structure provides the preliminary description of the atomic distribution. The tiny difference contains the information of the local disordered domains.










Figure S9. Lebail fitting for the XRD of Fe_3_Pt alloy. The fitting was conducted in the 2-theta range from 1 to 8.6°. In order to get the average lattice parameter, the XRD of the samples with partial chemical ordering were fitted by combining the ordered/disordered structures with the restrain of the same lattice parameters.


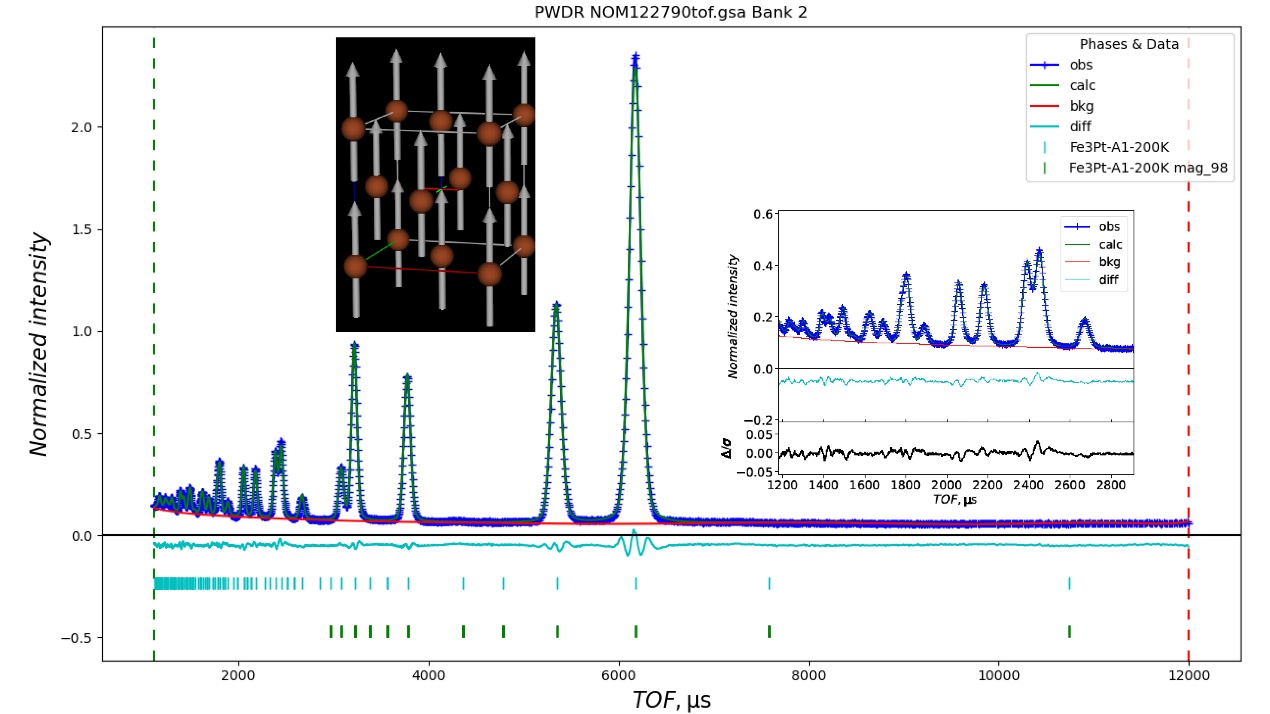


Figure S10. Rietveld refinements of TOF-mode neutron powder diffraction based on the disordered A1 phase with ferromagnetic structure of S1 utilizing GSAS-II. The insets show the ferromagnetic structure of disordered A1 phase and the zoom-in view of the high-Q area. After the refinement, the magnetic moment of crystal site is verified as 2.6308μ_B_.


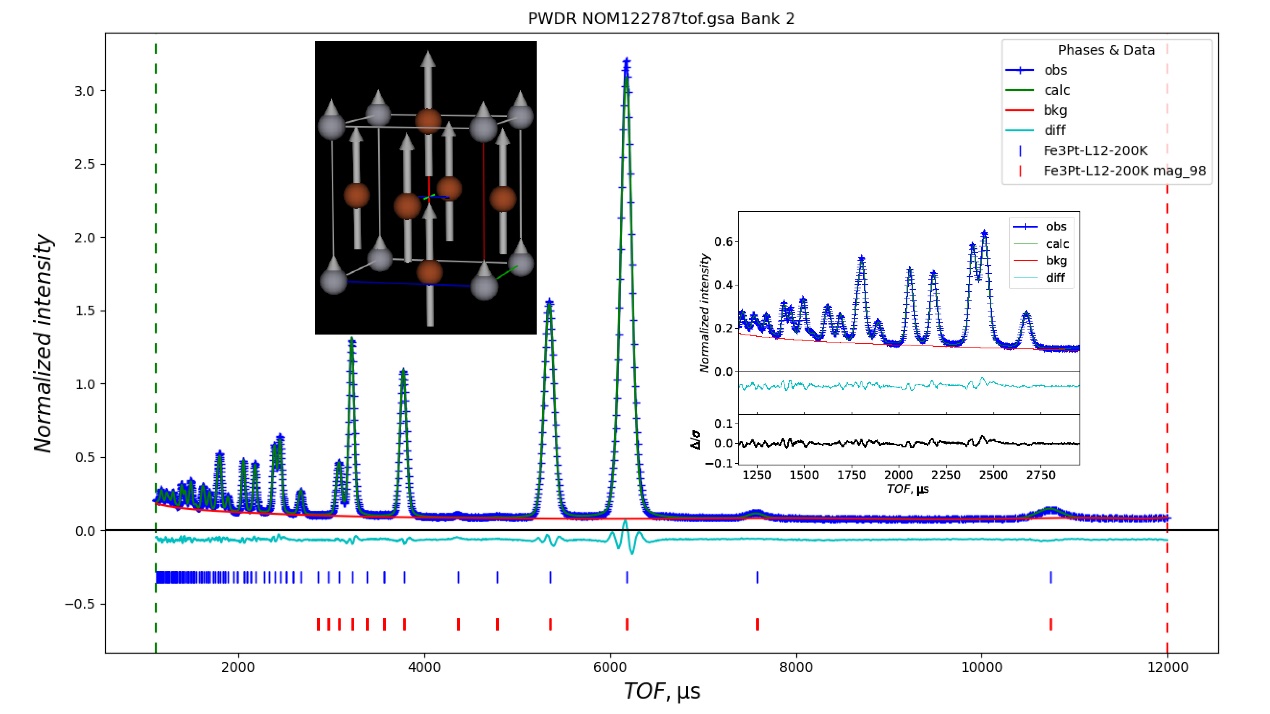


Figure S11. Rietveld refinements of TOF-mode neutron powder diffraction based on ordered L1_2_ phase with ferromagnetic structure of S3 utilizing GSAS-II. The insets show the ferromagnetic structure of ordered L1_2_ phase and the zoom-in view of the high-Q area. After the refinement, the magnetic moment of face-center sites is verified as 2.7959μ_B_ while the one on the corners is 0.9574μ_B_.

Table S1. Parameters from the decomposition of ^57^FeMössbauer spectra of Fe_3_Pt at -100°C.

|  | S3 | |
| --- | --- | --- |
|  | Line1 | Line2 |
| Component | 41.72% | 58.28% |
| Isomer shift(mm/s) | 0.30(1) | 0.32(1) |
| Quardrupole splitting(mm/s) | 0.03(1) | -0.01(1) |
| Effective magnetic field(T) | 32.6(1) | 34.4(1) |

Table S2. The calculated magnetic moment of disordered Fe_3_Pt alloy

|  | X | Y | Z | M(μ_B_) |  | X | Y | Z | M(μ_B_) |  | X | Y | Z | M(μ_B_) |
| --- | --- | --- | --- | --- | --- | --- | --- | --- | --- | --- | --- | --- | --- | --- |
| Fe | 0.1667 | 0 | 0.1667 | 2.77 | **Fe** | 0.5 | 0.5 | 0 | 2.72 | **Fe** | 0.6667 | 0.8333 | 0.1667 | 2.73 |
| Fe | 0.1667 | 0.1667 | 0 | 2.75 | **Fe** | 0.3333 | 0.3333 | 0.3333 | 2.79 | **Fe** | 0.8333 | 0.6667 | 0.1667 | 2.74 |
| Fe | 0 | 0 | 0.3333 | 2.66 | **Fe** | 0.5 | 0.3333 | 0.5 | 2.73 | **Fe** | 0.8333 | 0.8333 | 0 | 2.77 |
| Fe | 0 | 0.1667 | 0.5 | 2.62 | **Fe** | 0.5 | 0.5 | 0.3333 | 2.79 | **Fe** | 0.6667 | 0.8333 | 0.5 | 2.72 |
| Fe | 0.1667 | 0 | 0.5 | 2.7 | **Fe** | 0.3333 | 0.3333 | 0.6667 | 2.71 | **Fe** | 0.8333 | 0.6667 | 0.5 | 2.74 |
| Fe | 0.1667 | 0.1667 | 0.3333 | 2.69 | **Fe** | 0.3333 | 0.5 | 0.8333 | 2.67 | **Fe** | 0.8333 | 0.8333 | 0.3333 | 2.73 |
| Fe | 0 | 0 | 0.6667 | 2.67 | **Fe** | 0.5 | 0.3333 | 0.8333 | 2.69 | **Fe** | 0.6667 | 0.6667 | 0.6667 | 2.74 |
| Fe | 0 | 0.1667 | 0.8333 | 2.72 | **Fe** | 0.5 | 0.5 | 0.6667 | 2.7 | **Fe** | 0.8333 | 0.6667 | 0.8333 | 2.81 |
| Fe | 0.1667 | 0 | 0.8333 | 2.78 | **Fe** | 0.3333 | 0.6667 | 0 | 2.76 | **Fe** | 0.8333 | 0.8333 | 0.6667 | 2.78 |
| Fe | 0.1667 | 0.1667 | 0.6667 | 2.73 | **Fe** | 0.3333 | 0.8333 | 0.1667 | 2.81 | **Pt** | 0 | 0 | 0 | 0.19 |
| Fe | 0 | 0.5 | 0.1667 | 2.74 | **Fe** | 0.5 | 0.8333 | 0 | 2.78 | **Pt** | 0 | 0.1667 | 0.1667 | 0.22 |
| Fe | 0.1667 | 0.3333 | 0.1667 | 2.77 | **Fe** | 0.3333 | 0.8333 | 0.5 | 2.74 | **Pt** | 0 | 0.3333 | 0 | 0.19 |
| Fe | 0.1667 | 0.5 | 0 | 2.83 | **Fe** | 0.5 | 0.6667 | 0.5 | 2.78 | **Pt** | 0 | 0.5 | 0.5 | 0.19 |
| Fe | 0 | 0.3333 | 0.3333 | 2.7 | **Fe** | 0.3333 | 0.6667 | 0.6667 | 2.75 | **Pt** | 0.1667 | 0.5 | 0.3333 | 0.3 |
| Fe | 0.1667 | 0.3333 | 0.5 | 2.77 | **Fe** | 0.5 | 0.6667 | 0.8333 | 2.72 | **Pt** | 0 | 0.3333 | 0.6667 | 0.18 |
| Fe | 0.1667 | 0.3333 | 0.8333 | 2.77 | **Fe** | 0.6667 | 0 | 0 | 2.71 | **Pt** | 0 | 0.5 | 0.8333 | 0.24 |
| Fe | 0.1667 | 0.5 | 0.6667 | 2.81 | **Fe** | 0.6667 | 0.1667 | 0.1667 | 2.71 | **Pt** | 0 | 0.6667 | 0 | 0.17 |
| Fe | 0 | 0.8333 | 0.1667 | 2.71 | **Fe** | 0.8333 | 0 | 0.1667 | 2.75 | **Pt** | 0.1667 | 0.6667 | 0.1667 | 0.29 |
| Fe | 0.1667 | 0.8333 | 0 | 2.78 | **Fe** | 0.6667 | 0 | 0.3333 | 2.71 | **Pt** | 0 | 0.8333 | 0.5 | 0.15 |
| Fe | 0 | 0.6667 | 0.3333 | 2.74 | **Fe** | 0.8333 | 0 | 0.5 | 2.7 | **Pt** | 0 | 0.6667 | 0.6667 | 0.23 |
| Fe | 0.1667 | 0.6667 | 0.5 | 2.82 | **Fe** | 0.8333 | 0.1667 | 0.3333 | 2.7 | **Pt** | 0 | 0.8333 | 0.8333 | 0.2 |
| Fe | 0.1667 | 0.8333 | 0.3333 | 2.78 | **Fe** | 0.6667 | 0 | 0.6667 | 2.76 | **Pt** | 0.3333 | 0 | 0 | 0.19 |
| Fe | 0.1667 | 0.6667 | 0.8333 | 2.81 | **Fe** | 0.6667 | 0.1667 | 0.8333 | 2.71 | **Pt** | 0.3333 | 0.1667 | 0.5 | 0.14 |
| Fe | 0.1667 | 0.8333 | 0.6667 | 2.78 | **Fe** | 0.8333 | 0 | 0.8333 | 2.8 | **Pt** | 0.3333 | 0.5 | 0.1667 | 0.26 |
| Fe | 0.3333 | 0.1667 | 0.1667 | 2.67 | **Fe** | 0.8333 | 0.1667 | 0.6667 | 2.73 | **Pt** | 0.3333 | 0.5 | 0.5 | 0.2 |
| Fe | 0.5 | 0 | 0.1667 | 2.72 | **Fe** | 0.6667 | 0.5 | 0.1667 | 2.78 | **Pt** | 0.5 | 0.6667 | 0.1667 | 0.24 |
| Fe | 0.5 | 0.1667 | 0 | 2.69 | **Fe** | 0.8333 | 0.3333 | 0.1667 | 2.77 | **Pt** | 0.3333 | 0.6667 | 0.3333 | 0.27 |
| Fe | 0.3333 | 0 | 0.3333 | 2.7 | **Fe** | 0.8333 | 0.5 | 0 | 2.82 | **Pt** | 0.5 | 0.8333 | 0.3333 | 0.23 |
| Fe | 0.5 | 0 | 0.5 | 2.72 | **Fe** | 0.6667 | 0.3333 | 0.3333 | 2.69 | **Pt** | 0.3333 | 0.8333 | 0.8333 | 0.2 |
| Fe | 0.5 | 0.1667 | 0.3333 | 2.69 | **Fe** | 0.6667 | 0.5 | 0.5 | 2.69 | **Pt** | 0.5 | 0.8333 | 0.6667 | 0.22 |
| Fe | 0.3333 | 0 | 0.6667 | 2.73 | **Fe** | 0.8333 | 0.3333 | 0.5 | 2.74 | **Pt** | 0.8333 | 0.1667 | 0 | 0.28 |
| Fe | 0.3333 | 0.1667 | 0.8333 | 2.69 | **Fe** | 0.8333 | 0.5 | 0.3333 | 2.72 | **Pt** | 0.6667 | 0.1667 | 0.5 | 0.15 |
| Fe | 0.5 | 0 | 0.8333 | 2.74 | **Fe** | 0.6667 | 0.3333 | 0.6667 | 2.71 | **Pt** | 0.6667 | 0.3333 | 0 | 0.18 |
| Fe | 0.5 | 0.1667 | 0.6667 | 2.7 | **Fe** | 0.8333 | 0.3333 | 0.8333 | 2.85 | **Pt** | 0.6667 | 0.5 | 0.8333 | 0.18 |
| Fe | 0.3333 | 0.3333 | 0 | 2.69 | **Fe** | 0.8333 | 0.5 | 0.6667 | 2.78 | **Pt** | 0.6667 | 0.6667 | 0.3333 | 0.21 |
| Fe | 0.5 | 0.3333 | 0.1667 | 2.71 | **Fe** | 0.6667 | 0.6667 | 0 | 2.75 | **Pt** | 0.6667 | 0.8333 | 0.8333 | 0.17 |


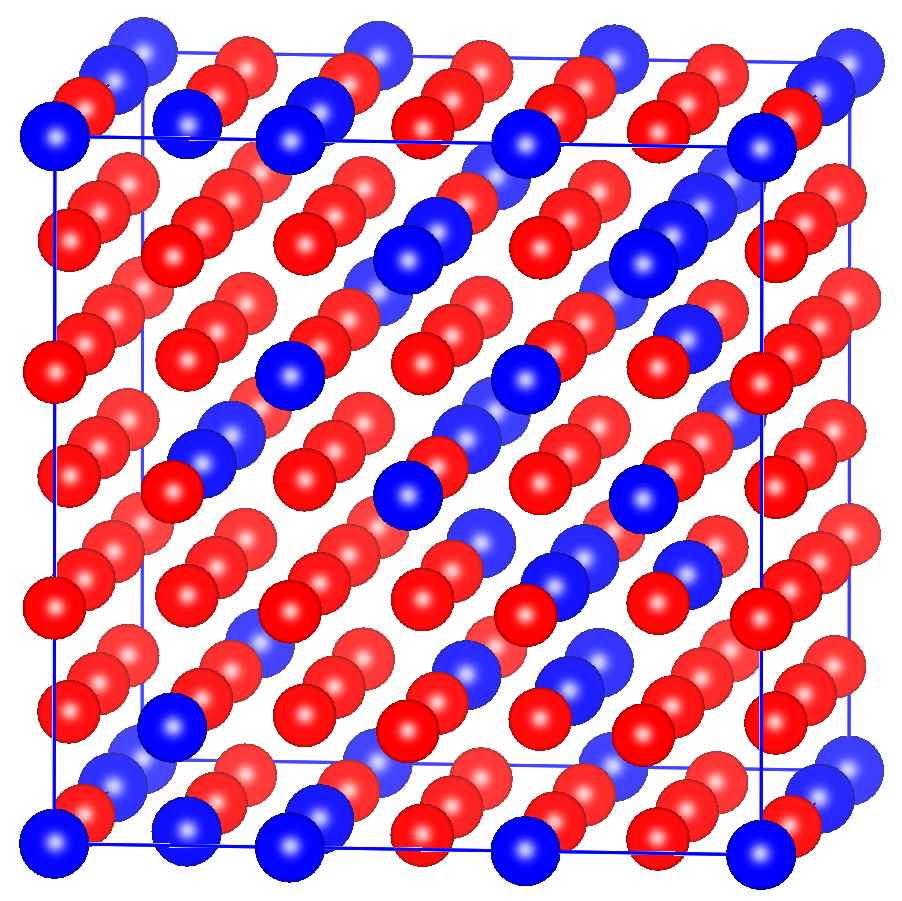

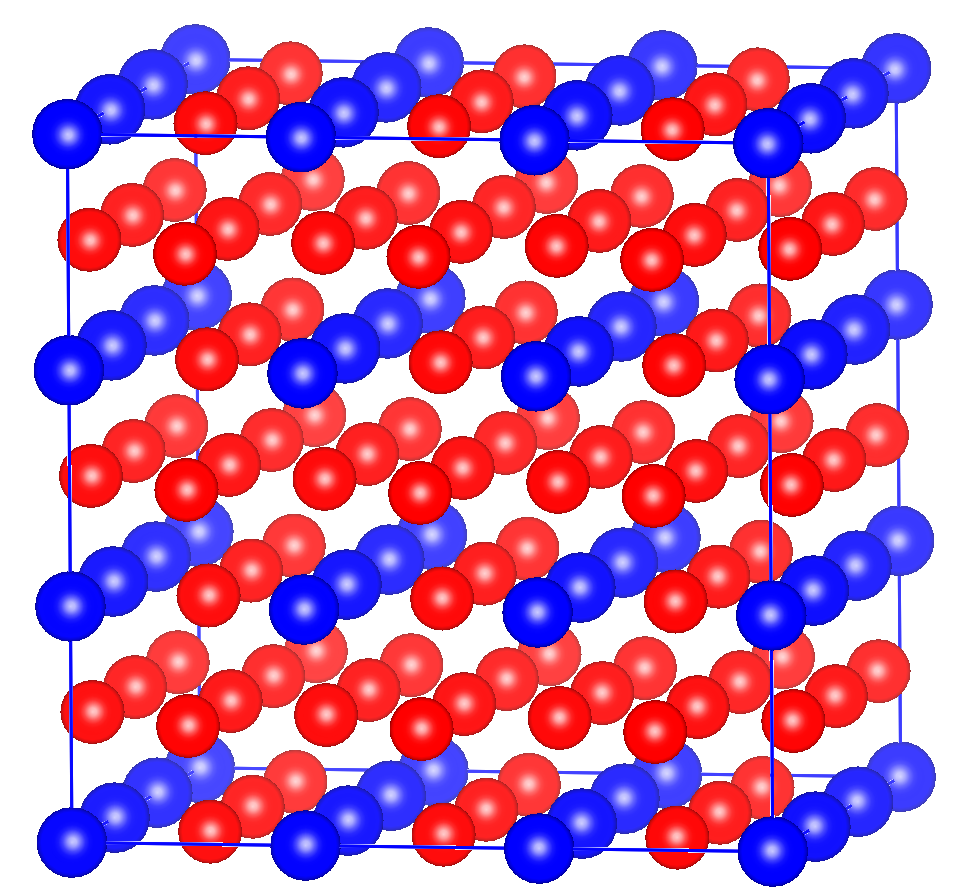


Figure S12. The models for first principles calculation. Left structure is the disordered model while right structure is the ordered one. According to the calculated results, the average Fe magnetic moment of disordered model is 2.734μ_B_ and the one of ordered model is 2.68μ_B_.
